# Secondary Cerebellar Cortex Injury in Albino Male Rats after MCAO: A Histological and Biochemical Study

**DOI:** 10.3390/biomedicines9091267

**Published:** 2021-09-18

**Authors:** Aziza R. Alrafiah

**Affiliations:** Medical Laboratory Technology Department, Faculty of Applied Medical Sciences, King Abdulaziz University, Jeddah 21589, Saudi Arabia; aalrafiah@kau.edu.sa; Tel.: +966-01264-01000 (ext. 23495); Fax: +966-01264-01000 (ext. 21686)

**Keywords:** middle cerebral artery occlusion, cerebellar cortex, angiogenic factors, secondary injury

## Abstract

The present study focused on secondary injury following the middle cerebral artery (MCA) occlusion in rats not linked to the MCA’s feeding zone. This entity has been very rarely studied. Additionally, this study investigated the rates of expression of five fundamental angiogenic biomarkers called endoglin, vascular endothelial growth factors-A (VEGF-A), endothelin-1 (ET-1), 2granulocyte colony-stimulating factor (G-CSF), and angiopoietin-using the MCA occlusion (MCAO) model. The random allocation of twelve adult male albino rats was in two groups. As a sham control group, six rats were used. This group was subjected to a sham operation without MCAO. The MCAO group consisted of six rats that were subjected to MCAO operation. After three days, the rats were sacrificed. The cerebellar specimens were immediately processed for light microscopic examination. An angiogenic biomarkers multiplex assay from multiplex was used to assess endoglin levels, VEGF-A, ET-1, angiopoietin-2, and G-CSF in serum samples. Hematoxylin and eosin-stained sections showed that the cerebellar cortex of rats of the MCAO group was more affected than the sham control group. Furthermore, Nissl stain and immunohistochemical analysis revealed an apparent increase in the number of positive immunoreactive in the cerebellar cortex and an evident decrease in Nissl granules in Purkinje cells of the MCAO rats, in contrast to the control rats. In addition, there was a significant increase in angiogenic factors VEGF-A, ET-1, angiopoietin-2, and endoglin. Interestingly, there was an increase in the G-CSF but a non-significant in the MCAO rats compared to the control rats. Furthermore, there was a significant correlation between the angiopoietin-2 and ET-1, and between G-CSF and ET-1. VEGF-A also exhibited significant positive correlations with the G-CSF serum level parameter, Endoglin, and ET-1. Rats subjected to MCAO are a suitable model to study secondary injury away from MCA’s feeding zone. Additionally, valuable insights into the association and interaction between altered angiogenic factors and acute ischemic stroke induced by MCAO in rats.

## 1. Introduction

Ischemic stroke is a severely impaired and high-incidence neurodegenerative condition. About 80% of all human strokes are ischemic strokes. They are caused by thrombotic and embolic occlusion that reduces or restricts blood flow in the MCA. The MCA is one of the main blood supplies in the brain [1].

Every year, about 15 million people suffer from strokes worldwide, according to the World Health Organization (WHO). Five million become permanently disabled, and five million die [2]. Stroke is becoming more and more of a critical health issue in the Middle East region, with the prediction that deaths caused by stroke will almost double by 2030 [3]. Stroke is recorded in Saudi Arabia as a rapidly increasing issue and a significant cause of disease and death. It is, therefore, one of Saudi Arabia’s most crucial social and economic problems [4].

Von Monakow invented the term “diaschisis”, defining it as the transient functional shock of regions distant from the lesion [5]. Various diaschisis patterns have been established. In 1981, in the presence of a hemispheric stroke, Baron et al. described crossed cerebral diaschisis (CCD) [6], a supratentorial lesion that causes hypoperfusion and hypometabolism in the contralesionally cerebellar hemisphere. However, the etiology of CCD is unknown [7]. To date, CCD mechanisms have been thought to start by interrupting the exiting input of the cerebellum, primarily through a cortico-pontocerebellar pathway [8,9].

The most focal model of brain ischemia is the occlusion of a significant brain vessel, such as the mid-cerebral artery (MCA). The MCAO means that brain flow in the striatum and cerebral cortex exhibited various reduction degrees, depending on the method. The occlusion of the MCA in rats usually leads to extensive neuronal cortex loss and caudal putamen damage that ranges from very little to extensive [1,10,11]. This model has pathological findings that are similar to those reported by human stroke victims.

One of the critical early events after cerebral ischemia is angiogenesis [12]. The animal models provide an opportunity to develop preventive strategies to reduce ischemic brain injury and restore severe impairment. The use of pro-angiogenic stimulation, in particular, is the chance to promote neurogenesis and reconstruction of ischemic brain areas that lack adequate collateral vasculature infusion. A deeper understanding of how pro-angiogenic impulses in the brain affect neuronal activity is necessary [13]. The VEGFs are the essential mediators of vasculogenesis and angiogenesis and show upregulation after occlusion of the MCA in rats. In addition, angiopoietin-2 has been identified as a vascular-specific receptor essential for vessel development [12]. Endothelial cells in cerebral blood vessels under the influence of astrocytes formed a blood–brain barrier (BBB). There is an increasing BBB permeability with humoral agents liberated by astrocytes, namely endothelin1 (ET-1) [14]. Lo and his colleagues suggested a role of ET-1 in BBB disruption in brain injury [15]. Granulocyte colony-stimulating factor (G-CSF) is a hematopoietic system endogenous peptide hormone currently undergoing phase I and II ischemic stroke clinical trials [16,17]. Several animal studies have shown that G-CSF has the potential to provide significant neuroprotection in brain ischemia, primarily because of high hormone-induced anti-apoptotic signals in the brain [18].

So, the current study aimed to focus on secondary injury following occlusion of the MCA in rats not linked to the feeding zone of the MCA. This entity has been very rarely studied. In addition, the purpose of this study was to investigate the rates of expression of five fundamental angiogenic biomarkers called angiopoietin-2, vascular endothelial growth factor-A (VEGF-A), endoglin, granulocyte colony-stimulating factor (G-CSF), and endothelin-1 (ET-1) using the MCA occlusion (MCAO) model.

## 2. Materials and Methods

### 2.1. Biochemicals

MULTIPLEX MAP Mouse Angiogenesis/Growth Factor Magnetic Bead Panel (Cat. # MAGPMAG-24K) was used for the quantification of any or all of the following analytes in serum: Angiopoietin-2 Bead (Cat. # MANGPT2-MAG), Endoglin Bead (Cat. # MENDGLN-MAG). Endothelin-1 Bead (Cat. # MET1-MAG), G-CSF Bead (Cat. # MGCSF-MAG), and VEGF-A Bead (Cat. # MVEGFC-MAG). All the kits were purchased from (Merck Millipore, Burlington, MA, USA).

### 2.2. Animals

Twelve adult male albino rats weighing 280–340 g at the beginning of the experiment. The animals were delivered to an animal house at King Fahd Medical Research Center, pharmacology department, Faculty of Medicine, King Abdulaziz University (KAU), Jeddah, Saudi Arabia. Animals were kept at a temperature of 23.2 °C, 12:12-h light/dark cycle, and were given free access to water and food in a group of six animals per cage. A seven-day adaptation period to the new environment was allowed before assigning subjects to the study groups. Restrictions to food (but not water) were applied 24 h before the MCA occlusion operation. All experiments were conducted during the light phase from 10:00 a.m. To 4:00 p.m.

### 2.3. Induction of Middle Cerebral Artery Occlusion (MCAO)

The intra-aluminum filament techniques, as described previously by Longa et al. [19], were used to develop an MCAO mode and modified to use an internal carotid artery (ICA) [20]. This simple technique is wholly noninvasive and was familiar to research neuroprotection mechanisms as well as cellular injury. The MCAO model includes the injection into the internal carotid artery (ICA) of a monofilament nylon suture 4–0, and then the cranial advancing of the suture. After 60 min (transient occlusion), the suture was removed.

### 2.4. Experimental Groups

The random allocation of twelve adult male albino rats was in two groups. As for the sham control group, six rats were used. This group underwent a sham operation. The MCAO group consisted of six rats subjected to MCAO operation. After three days, the rats were sacrificed by cervical dislocation under humane conditions, and the heads were dissected for obtaining the cerebellar biopsies. The specimens were immediately processed for light microscopic examination.

### 2.5. Blood Sample Collection and Preparation

From the ophthalmic venous plexus, the blood sample was collected through the retro-orbital approach. The blood was allowed to coagulate for at least 30 min before centrifugation to acquire serum aliquoted and stored at −20 °C and then used to calculate concentrations of angiogenic biomarkers. The procedure of measuring angiogenic biomarkers based on antigen–antibody reaction on immunoassay machines, called Luminex 200, is available in the central laboratory of the medical college at KAU.

### 2.6. Biochemical Measurements

MULTIPLEX^®^ MAP builds on Luminex^®^ xMAP^®^ technology—one of the most rapidly expanding and valued multiplex technologies that serves life science applications and bioassays. Luminex^®^ uses advanced methods to internally color-code microspheres of two fluorescent dyes internally color code. The exact quantities of these dyes may be used to create separately colored bead sets of 500 5.6 μm or 80 6.45 μm polystyrene microsphere, each protected by a different primary antibody. After an observer extracts the bead from the test sample, a biotinylated detection antibody is added. Streptavidin then incubates the reaction mixture to complete a reaction on the surface of each microsphere. In the presence of the 3.1 Xponent software package, the levels of Angiopoietin-2, Endoglin, Endothelin-1, G-CSF, VEGF-A in processed serum were calculated using Luminex technology.

### 2.7. Histological and Immune-Histochemical Studies for Light Microscopic Examination

In a 10% buffer paraformaldehyde, the cerebellum specimens were fixed for 48 h. The samples were dehydrated for 1 hour each in upward grades of alcohol (50%, 70%, 90%, and 95%). Next, two changes for one hour of absolute alcohol (100%) were done. After xylene clearing, we incorporated soft paraffin wax at 55 °C for 2 h, and then repeated the process for another 2 h using hard paraffin at 60 °C. Five μm thick sections were prepared for hematoxylin and eosin (H&E) staining. The stain of Nissl is used to stain Nissl substance in the cytoplasm of neurons. Additionally, the avidin-biotin-peroxidase technique was used to evaluate astrocytes glial fibrillary acidic protein (GFAP) counterstained with Hx. The positive reaction appeared as a brownish cytoplasmic reaction.

### 2.8. Statistical Analysis

Statistical analysis was conducted using the Statistical Package for the Social Science software package (SPSS) version 26.0 (IBM Corp., Armonk, NY, USA). All data were statistically analyzed using an unpaired student *t*-test. Correlations between measured parameters were made using Pearson correlations. Data are presented as mean ± standard deviation (S.D.). The significance was at ≤0.05. Graphs were made by GraphPad prism software version 8 (2019), San Diego, CA, USA.

## 3. Results

### 3.1. Histological Results

#### 3.1.1. Hematoxylin and Eosin (H&E)

##### Sham Control Group

**Figure 1:**
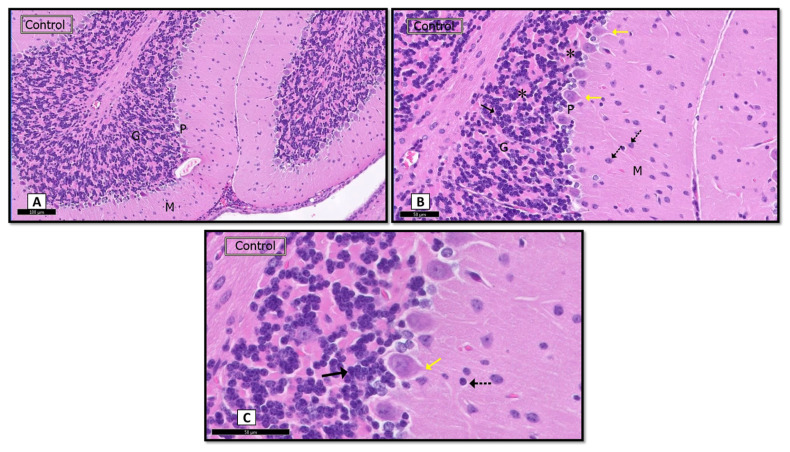
A section of a control rat showing: (**A**) the three layers; molecular (M), Purkinje cell (P), and granular cell layers (G). (H&E. ×10; Scale bar = 100 μm). (**B**,**C**) The Purkinje cells (P) in one row. Notice flask-shaped Purkinje cells with apical dendrites (yellow↑), pale stained nuclei, and prominent nucleoli. The granular layer appears consisting of closely packed rounded granule cells with rounded pale vesicular nuclei (Black arrow). Notice molecular layer (M) shows many glial cells (dot↑) and the spaces of cerebellar islands (*). (H&E. **B** × 20, **C** × 40; Scale bar = 50 μm).

Cerebellar cortex sections from H&E of the sham control group rats revealed that it formed in three consecutive layers. The molecular layer was on the outside, followed by the layer of Purkinje cells, whereas the granular layer was on the inside (Figure 1A). Purkinje cells appeared flask-shaped with apical dendrites. In one row, they were set. Characteristics of this were a pale basophilic cytoplasm and a central vesicular nucleus. Tightly packed granule cells and cerebellar islands appeared in the granular cell layer (Figure 1B,C).

##### MCAO Group

**Figure 2:**
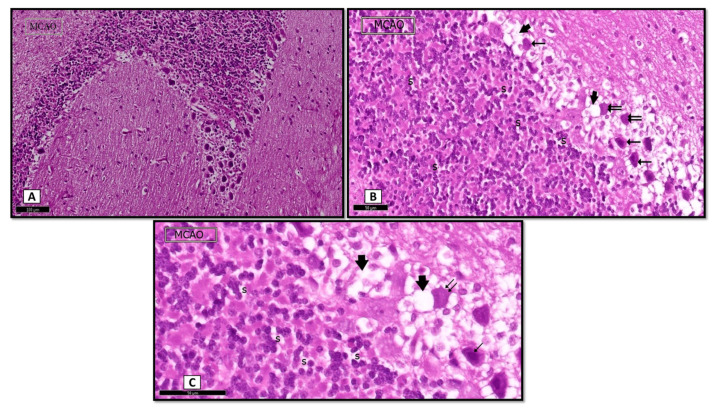
A section of MCAO rat showing: (**A**) the cerebellar cells of rats of the MCAO group shows destructive changes variable in severity and distribution. (**B**) Some Purkinje cells with homogenized cytoplasm and faint nuclei (↑↑). Notice vacuolation within the nearby molecular layer (Thick arrow). Granular layers show some spaces (s). (**C**) Higher magnification (H&E. **A** × 20, **B** × 40; Scale bar = 50 μm).

Stained sections with H&E showed that the cerebellar cells of rats of the MCAO group were more affected than the sham control group. Degenerated Purkinje cells were seen with darkly stained cytoplasm and nuclei. Additionally, halos were observed in empty spaces surrounding degenerated Purkinje cells. On the other hand, the granular layer cells were almost healthy, with some spaces between them (Figure 2A,B).

#### 3.1.2. Nissl Stain

**Figure 3:**
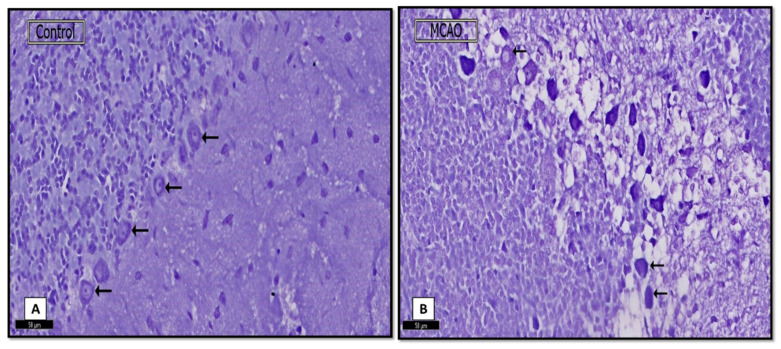
A Nissl-stained sections showing: (**A**) (control group) most of the Purkinje cells with Nissl’s granules (↑) in their cytoplasm. (**B**) (MCAO group) Some Purkinje cells with marked reduction of Nissl’s granules (↑) giving the faint cytoplasm appearance. (Nissl stain. ×20; Scale bar = 50 μm).

Purkinje cells’ cytoplasm appeared with an apparent increase in the Nissl’s granules surrounding central vesicular nuclei (Figure 3A). Interestingly, some Purkinje cells were lightly stained compared to the sham control group with an apparent reduction in their Nissl’s granules content (Figure 3B).

#### 3.1.3. Immunostained GFAP Sections

**Figure 4:**
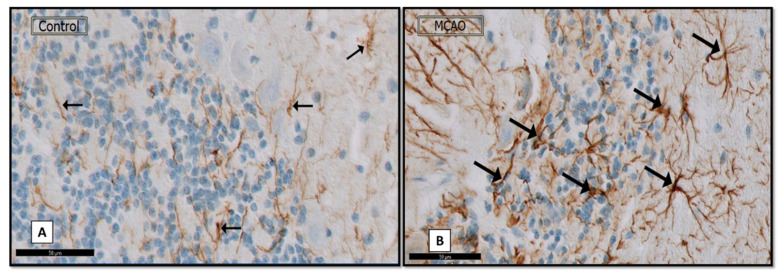
A section showing: (**A**) (control group) mild GFAP positive immunoreactive astrocytes (↑). (**B**) (MCAO group) strong GFAP positive immunoreactive astrocytes (↑). (GFAP × 40; Scale bar = 50 μm).

Immunostained sections of the sham control rats showed few GFAP positive immuno-positive astrocytes dispersed in the layers of the cerebellar cortex (Figure 4A). Interestingly, an apparent increase in the number of immunopositive cells in all layers contrasts with the shame control group (Figure 4B).

### 3.2. Biochemical Results

**Figure 5:**
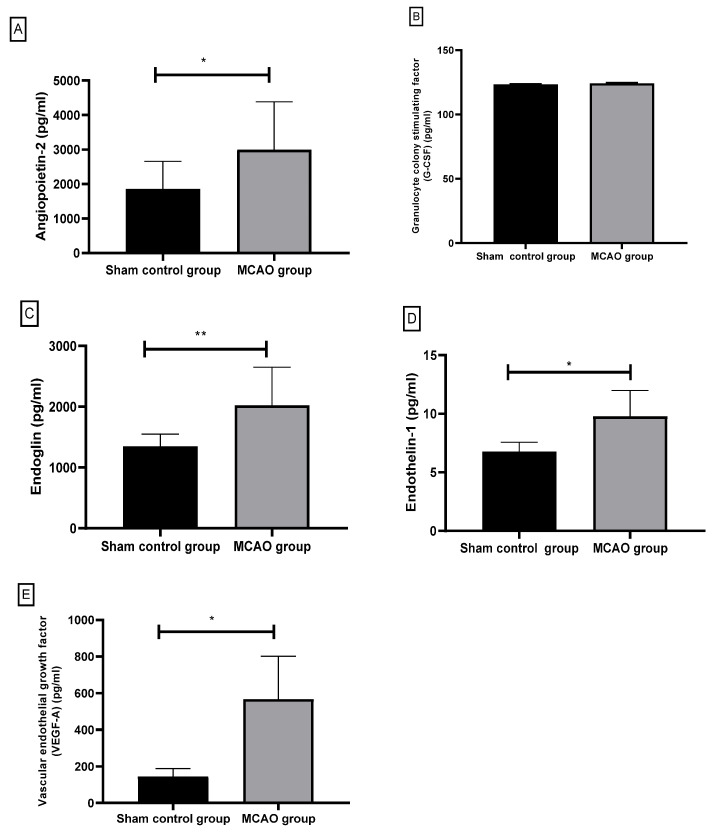
Comparison of angiogenic markers levels (**A**) Angiopoietin-2 (pg/mL), (**B**) Granulocyte colony-stimulating factor (G-CSF) (pg/mL), (**C**) Endoglin (pg/ml), (**D**) Endothelin-1 (ET-1) (pg/mL), (**E**) Vascular endothelial growth factor-A (VEGF-A) (pg/mL) between sham control group and middle cerebral artery occlusion (MCAO) group. Data are expressed as mean +/− standard deviation. * Significant at (<0.05) and ** significant at (<0.01) as compared to the sham control group.

In the current study, five fundamental angiogenic biomarkers named VEGF-A, angiopoietin-2, endoglin, endothelin-1, and G-CSF were investigated in the MCAO group versus the control group (Figure 5 and Table 1).

#### 3.2.1. Angiopoietin-2 (pg/mL)

In the current study, Angiopoietin-2 levels in the MCAO rats significantly increased (*p*-value < 0.05) versus the sham control rats, as shown in Figure 5A. Angiopoietin-2 level revealed concentrations of (2997.50 ± 1383.26 pg/mL) in the serum of the MCAO group and (1859.66 ± 798.30 pg/mL) for the sham control group.

#### 3.2.2. Granulocyte Colony-Stimulating Factor (G-CSF)

In this study, the G-CSF level exhibited a non-significant increase (*p* = 1.97) in the MCAO group versus the sham control group, as shown in Figure 5B. The G-CSF level concentrations (124.15 ± 0.71 pg/mL) for the MCAO group and the sham control group was (123.39 ± 0.51 pg/mL).

#### 3.2.3. Endoglin

In the current study, the serum level of Endoglin revealed a significant (*p* = 0.008) increase in the MCAO rats versus the control rats in Figure 5C. The Endoglin level concentrations (1349.50 ± 202.77 pg/mL) for the MCAO group and for the sham control group was (2025.70 ± 626.25 pg/mL).

#### 3.2.4. Endothelin-1 (ET-1)

In the current study, ET-1 levels in the MCAO rats significantly (*p* = 0.01) increased versus the sham control rats, as shown in Figure 5D. The ET-1 level revealed concentrations of (9.78 ± 2.19 pg/mL) in the serum of the MCAO group and (6.76 ± 0.80 pg/mL) for the sham control group.

#### 3.2.5. Vascular Endothelial Growth Factor-A (VEGF-A)

In the present study, the VEGF-A level exhibited a significant increase (*p* = 0.02) in the MCAO rats versus the sham control rats, as shown in Figure 5E. The VEGF-A level concentrations (566.68 ± 235.12 pg/mL) for the MCAO group and for the sham control group was (144.12 ± 44.50 pg/mL).

#### 3.2.6. Pearson’s Correlation between Angiogenesis Markers among the Groups

**Figure 6:**
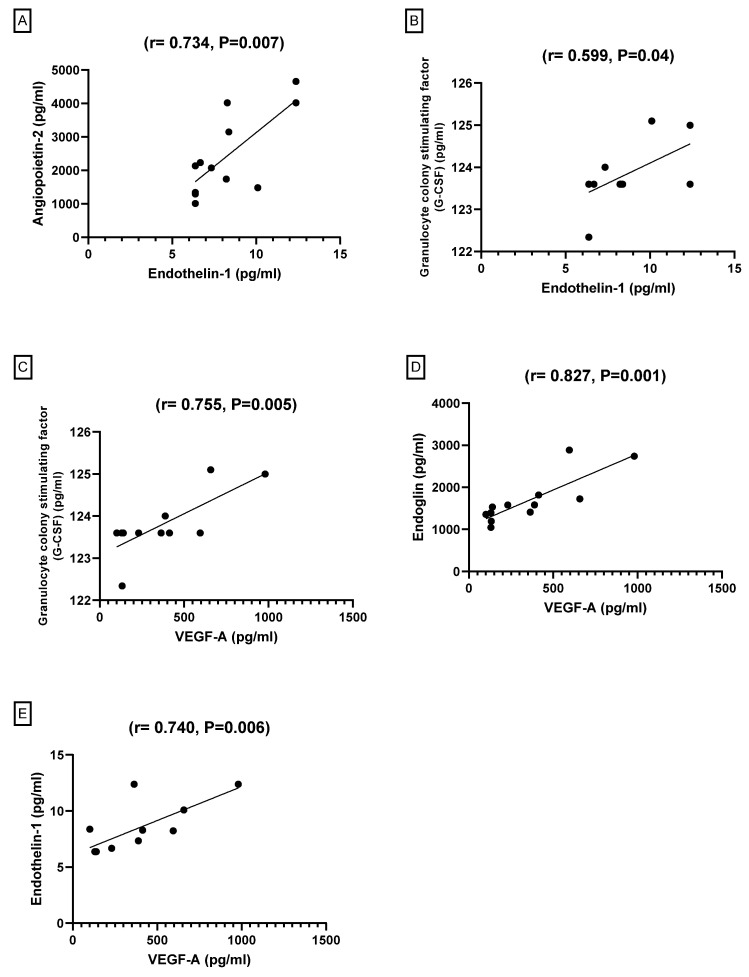
Correlations between Endothelin-1 and angiopoietin-2 (**A**) and G-CSF (**B**). Correlations between VEGF-A and Endothelin-1 (**C**), endoglin (**E**), G-CSF and (**D**).

In the present study, the Pearson correlation test for all correlations among all study groups was used. Table 2 and Figure 6 showed a significant positive correlation between Angiopoietin-2 and ET-1 (r = 734, *p* = 0.007). Interestingly, the comparison groups showed significant positive correlations between G-CSF and ET-1 in the serum level parameter (r = 0.599, *p* = 0.04). In addition, VEGF-A also exhibited significant positive correlations with the G-CSF serum level parameter (r = 0.755, *p* = 0.005), Endoglin (r = 0.827, *p* = 0.001), and ET-1 (r = 0.740, *p* = 0.006).

## 4. Discussion

Stroke is the most debilitating condition for human wellbeing. Various animal models were used in previous studies to mimic ischemic human stroke with different occlusion intervals [21]. In the present study, intra-aluminum filament techniques were used to induce the MCAO model in albino rats. This method results in an interference with blood flow to the MCA from ICA. MCAO is a useful experimental model for focal cerebral ischemia, which causes chronic infarction in the areas supplied by it [22].

For ischemic brain lesions, the focus is not on the secondary injury, which has recently attracted researchers’ attention, but only on the clinical and histological improvements at the site of ischemia. Other studies have indicated that neurological trauma to the brain was not limited to a regional infarction but also contributed to exofocal post-ischemic neuronal deaths [22,23]. According to Zhao et al., the MCAO rat’s substantia nigra was losing healthy neurons and increasing astrocyte proliferation [24]. In the spinal cord, hippocampus, hypothalamic, and substantia nigra pars reticulata regions following focal cerebral ischemic lesions, secondary changes in the form of activated microglia and astrocytes were also observed [25,26,27].

In this study, cerebellar changes were observed for rats following MCAO. The results of H&E stained cerebellar sections exhibited degenerative histological changes in the MCAO group. Similar findings were observed by [7,23]. Jie and his colleagues found apoptosis of neural cells of the cerebellar cortex in the MCAO group by the electron transmission microscope and further confirmed the results by TUNEL assay. They reported that the apoptotic cell count was increasing dynamically in MCAO compared to the control group by the time of the MCAO. They explained that the apoptosis mechanism of the cerebellar cortex neural cells was triggered after MCAO, probably due to the disruption of the pathways of the nerves.

A consequence of a neuronal injury is a simple disruption to neural cells and axonal degeneration. The apoptosis of cerebellar cortex neural cells caused by MCAO may be linked to the disruption of cortical pathways [23]. In comparison, some authors observed and regarded shrunk Purkinje cells with darkly stained cytoplasm and undefined nuclei as a sign of chromatolysis and gliosis. Rapid response to brain damage that is mediated by soluble neuron mediators or cell–cell interaction may be clarified by gliosis or decreased neuralgic cells. Other researchers considered dark shrunk neurons as a mirror with a much-condensed cytoplasm and nucleoplasm [28,29].

The cytoplasm of Purkinje cells in Nissl stained cerebellum areas from the MCAO group showed that some Purkinje cells were stained slightly compared to the sham control group, where the content of Nissl’s granules was apparently decreased. The process is called chromatolysis, which Hanz and Fainzilber explained [30]. They stated that a neuronal cytoplasm reaction occurs following traumatic or metabolic damage.

Regarding the cytoplasmic immune reaction of the GFAP in the astrocyte cell body and the processes thereof, there was a significant increase in contrast with the sham control group in the MCAO rats. GFAP is the mature astrocyte intermediate filament; after, it is known as specific astrocyte markers [31]. Published investigations suggested that any degenerative brain injury could trigger the proliferation of astrocytes and hypertrophy, resulting in severe astrogliosis with increased GFAP positive reactions. The presence of these neuronal astrocytes as a result of neurodegeneration can be seen as a compensatory mechanism. Such activated astrocytes benefit tissue repair after injury by tracking pH, ion homeostasis, and extracellular fluid. Astrocytes, through over-activation, can, however, lead to adverse effects [29,32].

The selection of the growth factors evaluated in the present study (angiopoietin-2, VEGF-A, and G-CSF) was performed due to previous pre-clinical models of cerebral ischemia [33,34]. In this study, there was a significant increase in the serum level of the growth factors angiopoietin-2 and VEGF-A in the MCAO group compared to the sham control group. There was an increase in G-CSF serum level in the MCAO rats but this was non-significant when compared to the control rats. In addition, there were significant positive correlations between VEGF-A and G-CSF. Similar findings were observed by [35]. Sobrino and his colleagues reported that the serum level of VEGF, CF, Ang-1, and stromal-related factor-1α showed a similar biological trend with maximum values at day seven and remained higher in the first three months [35].

G-CSF promotes the mobilization of stem cells from the bone marrow into compromised brain areas, reducing infarction and increasing plasticity and vascularization of the neuron [36,37,38]. The G-CSF showed a slight enhancement in function but not in neurological impairment [33]. While Ang-1 may be the interaction between neurogenesis and angiogenesis, it facilitates neuron migration and functional recovery after ischemic stroke [35,39,40]. Similar findings were observed by Zhang and his colleagues, who reported that there was a significant increase in serum level of the angiopoietin-1 in the mice model of the ischemic brain induced by embolic MCAO. They explained that angiopoietin-1 reduces BBB leakage and consequently decreases ischemic lesion volume [41]. After the ischemic injury, VEGF and angiopoietin-1 play a significant role in angiogenesis [42]. Angiopoietin-1 supports angiogenesis and new vessel remodeling. It is also an anti-permeability factor and recruits vascular smooth muscle cells and pericytes to surround endothelial cells so that new blood vessels are eventually developed and stabilized [43].

The VEGF family includes five members for primates, including Placental Growth Factor and VEGF-A/B/C/D.

The VEGF-A molecule is exceptional in this family due to its ability to facilitate neuroprotection and angiogenesis [44,45,46,47,48]. VEGF is a central angiogenesis regulator in an animal stroke model. Increasing vessel density in the peri-infarct zone follows VEGF expression [18,49,50,51,52,53]. Furthermore, VEGF causes astrocyte trans-differentiation into new neurons after acute ischemic strokes and, thus, facilitates neurogenesis [54]. In the setting of acute ischemic stroke, it makes VEGF-A’s supportive role in both angiogenesis and neurogenesis an attractive molecular objective for rational drug therapy. Under these conditions, in the early recovery phase following acute ischemic stroke, the rise in endogenous VEGF concentrations and the use of exogenous VEGF in ischemic brains are potentially injurious [55]. The dual role of VEGF-A in acute ischemic stroke is by creating an endothelial network by enhancing endothelial cell migration and proliferation, and it also induces vascular leakage and permeability [56]. Additionally, a recent research study in a rodent MCAO model of acute ischemic stroke by Zhang and his colleagues stated that upregulation in the matrix metalloproteinases (MMPs) could contribute to cerebrovascular edema caused by VEGF-A and inflammatory disruption in the acute ischemic stroke. They also concluded that early VEGF secretion would contribute to endothelial leakage after acute ischemic stroke, premature inhibition of VEGF, and a decrease in neurovascular permeability with decreased neurological function. They also stated that these neuroprotective effects would decrease rates of MMP 2 and 9 and, at the same time, increase tight junction proteins such as occludin [53]. Therefore, VEGF and Ang-1 exert synergistic effects on angiogenesis after stroke [57]. Lee et al. found that higher levels of VEGF-A lead to improving results in stock recovery, contrary to this research [58]. Furthermore, Matsuo et al. found that the VEGF-A concentration correlated positively to the severity of the stroke in cardioembolic infarction and correlated negatively with the severity of the extent of the atherothrombotic infarction [59].

Endoglin is an essential molecule in physiological and pathological vascular changes. Endoglin is the most critical element in endoglin-deficient mouse angiogenesis and vascular remodeling studies. Endoglin is present in Endothelial cells with relatively low levels of physiological conditions, but expression increases in Endoglin during angiogenesis and vascular damage [60]. VEGF-A induces endothelial activation by binding to vascular endothelial growth factor receptor two and subsequent leukocyte extravasation. In addition, endoglin expression is linked to inflammation in the endothelial function [61,62]. This could explain why there was a significant positive correlation between VEGF-A and endoglin in the present study.

Endothelin-1 can be a potential biomarker for BBB dysfunction as ET-1 overexpression contributes to further deposition of fluid and brain edema in experimental models following MCAO [63]. In acute phases, ET-1 controls the endothelial function that mediates vasoconstriction or vasodilation depending on the receptor, and contributes to increased permeability of the BBB [64,65]. In addition, high levels of ET-1 serum were found in ischemic patients [63,65]. This could explain why the ET-1 serum in the MCAO group was significantly higher than in the control group.

## 5. Conclusions

In conclusion, the secondary injury of the cerebellar cortex could be investigated by using middle cerebral occlusion in rats as an example. In addition, in the present study, prominent findings revealed valuable insights into the association and interaction between altered levels of angiogenic factors and acute ischemic stroke induced by middle cerebral artery occlusion in rats. Therefore, with the generation of significant results, it seems necessary to routinely monitor the angiogenic factor levels during acute ischemic stroke to decide proper intervention to stop the harmful outcomes of cerebral stroke and neurological deficiency.

## Figures and Tables

**Table 1:** Comparison of angiogenic factors between control rats and middle cerebral artery occlusion rats.

	Group	N	Mean ± Std. Deviation	Significance
Angiopoietin-2 (pg/mL)	sham control group	6	1859.66 ± 798.30	0.013 *
	MACO group	6	2997.50 ± 1383.26	
Granulocyte colony stimulating factor (G-CSF) (pg/mL)	sham control group	6	123.39 ± 0.51	0.197
	MACO group	6	124.15 ± 0.71	
Endoglin (pg/mL)	sham control group	6	1349.50 ± 202.77	0.008 **
	MACO group	6	2025.70 ± 626.25	
Endothelin-1 (ET-1) (pg/mL)	sham control group	6	6.76 ± 0.80	0.01 *
	MACO group	6	9.78 ± 2.19	
VEGF-A (pg/mL)	sham control group	6	144.12 ± 44.50	0.02 *
	MACO group	6	566.68 ± 235.12	

Values are set as mean ± S.D. Significance between sham control group and MCAO group using unpaired student *t* test. * Significant at (<0.05), ** significant at (<0.01).

**Table 2:** Pearson correlations between angiogenesis markers among the two groups of the study (sham control group and MCAO group).

		Angiopoietin-2 (pg/mL)	G-CSF (pg/mL)	Endoglin (pg/mL)	Endothelin-1 (ET-1) (pg/mL)
G-CSF (pg/mL)	Pearson Correlation	0.303			
	Sig. (2-tailed)	0.339			
Endoglin (pg/mL)	Pearson Correlation	0.344	0.474		
	Sig. (2-tailed)	0.274	0.120		
Endothelin-1 (ET-1) (pg/mL)	Pearson Correlation	0.734 **	0.599 *	0.462	
	Sig. (2-tailed)	0.007	0.040	0.131	
VEGF-A (pg/mL)	Pearson Correlation	0.472	0.755 **	0.827 **	0.740 **
	Sig. (2-tailed)	0.121	0.005	0.001	0.006

The correlation coefficient was made using the Pearson test. Middle cerebral artery occlusion (MCAO), Granulocyte colony-stimulating factor (G-CSF), Vascular endothelial growth factor-A (VEGF-A). ** Correlation is significant at the 0.01 level (2-tailed). * Correlation is significant at the 0.05 level (2-tailed).

## Data Availability

The data used to support the findings of this study were included within the article.
